# Genetics of Resistance to the Rust Fungus *Coleosporium ipomoeae* in Three Species of Morning Glory (*Ipomoea*)

**DOI:** 10.1371/journal.pone.0028875

**Published:** 2011-12-14

**Authors:** Thomas M. Chappell, Mark D. Rausher

**Affiliations:** Department of Biology, Duke University, Durham, North Carolina, United States of America; Emory University School of Medicine, United States of America

## Abstract

We examined the genetic basis of resistance to the rust pathogen *Coleosporium ipomoea* in three host species: *Ipomoea purpurea*, *I. hederacea*, and *I. coccinea* (Convolvulaceae). In crosses between resistant and susceptible individuals, second-generation selfed offspring segregated in ratios that did not differ statistically from the 3∶1 ratio indicative of single-gene resistance with the resistant allele dominant. One out of three crosses between resistant individuals from two different populations revealed that resistance loci differed in the two populations, as evidenced by the production of susceptible individuals among the S_2_ generation. These results suggest that gene-for-gene interactions contribute substantially to the dynamics of coevolution in this natural pathosystem. They also suggest that evolution of resistance to the same pathogen strain may involve different loci in different *Ipomoea* populations.

## Introduction

Gene-for-gene interactions are characteristic of plant-pathogen interactions in many agricultural systems [1], [2]. Such interactions are characterized by a number of properties: the ability of a particular pathogen strain to infect a particular crop variety is determined by genotype at one locus in the host and one locus in the pathogen; resistant plants typically exhibit a hypersensitive response to pathogens, which involves localized cell death surrounding the point of pathogen entry into plant tissue; and the apparent absence of both a universally virulent pathogen strain and a universally resistant plant genotype [3]–[5].

In many agricultural systems, gene-for-gene interactions give rise to a form of coevolution involving humans, a crop, and its pathogens. Humans introduce a novel resistance allele into a crop population, which provides protection from the current pathogen strains for up to a period of years. Eventually, however, a mutation conferring new virulence arises and spreads through the pathogen population, leaving the crop once again susceptible to the pathogen. In response, breeders again introduce a novel resistance mutation and the cycle repeats itself [6].

Because of the widespread occurrence of this type of coevolutionary dynamic in agricultural systems, it has been suggested or assumed that coevolution between plants and pathogens in nature is often also governed by gene-for-gene mediated interactions [6]–[8]. However, insufficient empirical evidence exists to distinguish between this hypothesis and the alternative that coevolution in nature primarily involves resistance and virulence that are inherited in a quantitative manner [9]–[11], In only a few natural systems have genetic analyses been undertaken to detect gene-for-gene interactions [*e.g.* 12]–[17], precluding an assessment of how frequently such interactions underlie coevolution in nature.

We report here a genetic analysis of resistance in several species of *Ipomoea* (morning glories) to infection by the rust pathogen *Coleosporium ipomoeae*. In southeastern North America, *I. purpurea*, *I. hederacea*, and *I. coccinea* are frequently infected by this rust. However, some individuals appear to be resistant in that they do not develop orange lesions characteristic of infection but do exhibit small patches of necrotic tissue that are characteristic of a hypersensitive response. Previously it has been shown that resistance to one rust strain in populations of *I. purpurea* is consistent with single-gene inheritance; in addition, complementation tests failed to demonstrate that different genes contributed to resistance in different populations [18].

Here we extend this analysis to additional rust strains and to additional host species. A recent investigation of host and rust populations in North Carolina revealed extensive between-population variation in both host resistance to particular rust strains and rust virulence on particular host species [19]. In particular, among 12 rust strains tested there were 11 unique patterns of infection across 13 combinations of host species and site of origin. Similarly, there were at least 3, 3, and 4 distinct resistance genotypes among 4,4, and 5 populations of *I. coccinea*, *I. purpurea*, and *I. hederacea* tested, as judged by patterns of infectivity by the 12 rust strains. Most individual rust strains could infect multiple host species, but not all populations of those host species. These patterns suggest that there is extensive coevolution occurring in this region between the rust and the three host species. Through a series of crosses between susceptible and resistant plants identified by this previous study, we demonstrate that inheritance of resistance to a particular rust strain is consistent with resistance being controlled by a single locus, with the resistant allele dominant. In addition we report the results of allelism tests designed to determine whether resistance in different populations is controlled by different genes.

## Results

In none of the crosses between resistant and susceptible populations (Table 1A) did the ratio of resistant to susceptible S_2_ individuals differ significantly from the expected 3∶1 ratio (Table 2). In each cross, none of the individual pairs showed significant deviation from this ratio and the test of heterogeneity among pairs was never significant. Finally, in all four crosses, the ratios after pooling across pairs was very close to 3∶1 and did not deviate significantly from this ratio. Thus, in each case, inheritance of resistance is consistent with resistance being determined by a dominant allele at a single locus.

**Table 1:** Crosses performed and rust accession used to test for resistance/susceptibility.

A. Tests for genetic basis of resistance			
Cross	Species	Host accessions crossed	Rust accession
**1**	*I. purpurea*	CRG-P (S)×CL-P (R)	CRG-P
**2**	*I. purpurea*	CRG-P (S)×LF-P (R)	CRG-P
**3**	*I. hederacea*	CRG-H (S)×LF-H (R)	LF-P
**4**	*I. coccinea*	CRG-C (S)×MO-C (R)	MO-H

B. Allelism Tests			
Cross	Species	Host accessions crossed	Rust accession
**5**	*I. hederacea*	CRG-H (R)×LF-H (R)	CRG-C
**6**	*I. purpurea*	CL-P (R)×LF-P (R)	CRG-P
**7**	*I. purpurea*	CL-P (R)×LF-P (R)	Ellis-P

(S) designates susceptible and (R) designates resistant.

**Table 2:** Cross results: tests for deviation from single locus inheritance.

Cross	Parental Pair	No. R S_2_	No. S S_2_	Ratio R/S	LR χ^2^
**1**	1	41	13	3.15 ∶ 1	0.0247
	2	39	14	2.79 ∶ 1	0.0566
	3	46	17	2.71 ∶ 1	0.1323
	4	32	16	2.63 ∶ 1	0.2069
	5	51	17	3.00 ∶ 1	0
	6	40	14	2.86 ∶ 1	0.0247
	heterogeneity				0.0013
	pooled	259	91	2.85 ∶ 1	0.0009
**2**	1	52	17	3.06 ∶ 1	0.0048
	2	50	16	3.13 ∶ 1	0.0202
	3	45	17	2.65 ∶ 1	0.1935
	4	37	13	2.85 ∶ 1	0.0267
	5	41	15	2.73 ∶ 1	0.0952
	6	42	19	2.21 ∶ 1	1.2295
	heterogeneity				0.0047
	pooled	267	97	2.75 ∶ 1	0.0025
**3**	1	68	21	3.24 ∶ 1	0.0936
	2	64	25	2.56 ∶ 1	0.4532
	3	69	23	3.00 ∶ 1	0
	4	60	21	2.86 ∶ 1	0.037
	heterogeneity				0.0025
	pooled	261	90	2.90 ∶ 1	0.0004
**4**	1	31	9	3.44 ∶ 1	0.1333
	2	36	11	3.27 ∶ 1	0.0638
	3	29	9	2.22 ∶ 1	0.0351
	4	18	7	2.57 ∶ 1	0.12
	5	22	10	2.20 ∶ 1	0.6667
	6	35	12	2.92 ∶ 1	0.0071
	7	18	7	2.57 ∶ 1	0.12
	8	30	12	2.50 ∶ 1	0.2857
	heterogeneity				0.0073
	pooled	219	77	2.84 ∶ 1	0.0009

No. R S_2_: number of S_2_ that were resistant. No. S S_2_: number of S_2_ that were susceptible. Ratio R/S: ratio of resistant to susceptible individuals. LRχ^2^: value of likelihood ratio chi-square statistic. None were significant. Critical value for 1 d.f. is 3.84 at P<0.05. Heterogeneity: test for heterogeneity among Parental Pairs for segregation ratios. Pooled: test for deviation from 3∶1 ratio after pooling S_2_'s from all Parental Pairs.

In the allelism tests (Table 1B), neither of the crosses involving *I. purpurea* populations exhibited any susceptible individuals (Table 3). The estimated recombination rate is therefore 0 in each case. However, even though more than 100 individuals were scored in each cross, the power to detect non-allelism between linked loci is low. This is reflected in the confidence interval for recombination rate, r, which indicates that the data are consistent with the results we expect under the hypothesis of there being different but moderately linked resistance loci in the two populations. By contrast, in the cross involving *I. hederacea*, 11 susceptible individuals were detected, indicating that different loci are responsible for resistance in the two populations tested. Using a significance criterion of P<0.05, the two loci are significantly linked (Table 3), although the upper bound on r of 0.49 is very close to absence of linkage.

**Table 3:** Numbers of resistant and susceptible plants in allelism tests.

Cross	Host Species	Rust Accession	No. Resistant Individuals	No. Susceptible Individuals	r (confidence interval)
**5**	*I. hederacea*	CRG-C	297	11	0.37 (0.27, 0.49)
**6**	*I. purpurea*	CRG-P	122	0	0.00 (0, 0.32)
**7**	*I. purpurea*	Ellis-P	155	0	0.00 (0, 0.28)

## Discussion

Our results suggest that gene-for-gene interactions between *Ipomoea* species and the pathogenic rust *Coleosporium ipomoeae* are an important component of the coevolutionary interactions among these species. In particular, we find that in all four combinations of host and pathogen accessions examined, resistance to the rust appears to be determined by segregation at a single locus with two alleles. Previously, an additional cross involving accessions different from those examined here also yielded similar results [18]. Although these results do not preclude the possibility that quantitative variation for resistance may also exist in this system, they do suggest that gene-for-gene interactions are common. One possible caveat to this conclusion is that we have not genetically characterized variation in virulence in the rust because techniques for crossing the sexual stages have not been developed. However, virulence in *C. ipomoea* exhibits the typical all-or-none pattern across host accessions that are typically associated with gene-for-gene interactions [19].

Another potential limitation of our analyses is that we used spore suspensions that may have contained multiple rust genotypes, rather than single-spore isolates. However, although there may have been more than one genotype in our suspensions, they all appear to react similarly to the resistance/susceptibility factors present in the host plants against which they were tested. If this were not true, it would have been very unlikely that we would have seen the clear 3∶1 ratios of resistant to susceptible plants that we obtained. Similarly, mixtures of genotypes with different compatibilities are inconsistent with the results of the test that revealed different resistance loci in different populations. Suppose, for example, the suspension used in Cross 5 of Table 1B was a mixture of two rust genotypes: one can overcome resistance conferred by allele R_1_ in host population CRG-H, but cannot overcome resistance conferred by allele R_2_ in host population LF-H; the other can overcome resistance produced by allele R_2_ in host population LF-H, but not resistance due to R_1_ in host population CRG-H. In this case, inoculation would infect minimally 3/8 of the F_2_ individuals (no linkage between loci) and up to 1/2 of those individuals (complete linkage). These proportions are far greater than the 3.57 percent of F_2_ individuals that were susceptible (Table 3). Additionally, the resistance response in this system is not cryptic, and plants challenged with mixtures of virulent and avirulent rust accessions responded with both infection and the hypersensitive response (data not shown). No cross-inoculations using single accessions produced this mixed result.

In all three host species examined, resistant plants exhibited a typical hypersensitive response, as recognized by small patches of necrotic tissue that develop upon exposure to the pathogen. In other plant species, resistance genes associated with the hypersensitive response are R-genes, which often encode proteins involved in the detection of pathogens or pathogen activity [20]–[22]. Moreover, R-genes form the basis of most characterized gene-for-gene interactions [1], [22]–[24]. Whether the interactions we observe are indeed determined by R-genes will require further characterization of these interactions at the molecular level.

In two of our four test crosses (Table 1A), single-gene resistance prevents infection by a strain of rust that occurs naturally at the site of the resistant population. This result is consistent with the hypothesis that this resistance has been selected for by this and other co-occuring rust strains, especially since infection by this rust pathogen substantially reduces host fitness [25]. By contrast, in the other two crosses, host populations that do not normally encounter the rust strain used carry single-gene resistance to that rust strain. One possible explanation of this apparently “pre-existing” resistance is that it has evolved due to selection imposed by other, local rust strains, and that the rust strain used in the test is not able to overcome this resistance because it has never encountered it, and thus has never been subjected to selection to overcome it. Even if this explanation is not true, though, it is clear that this type of pre-existing resistance has the potential to prevent successful establishment by dispersing rust strains, and thus to influence the dynamics of coevolution.

In other plant species, R-genes occur in multigene families consisting of tens or hundreds of copies, and these copies are typically organized into several unlinked clusters, each containing many tandemly repeated copies [26]. This pattern suggests that gene-for-gene coevolution may often involve the fixation of alleles resistant to a particular pathogen strain at different loci in different populations. Our results support this expectation. In particular, in one out of three allelism test crosses, in two resistant populations, alleles at different loci conferred resistance to the same pathogen accession. Furthermore, the lack of recombinant susceptible individuals in the other two test crosses does not preclude the possibility that different loci were involved, since different loci within the same tandem-repeat cluster would have very low recombination rates that would preclude detection of recombinant susceptibles with the sample sizes used here.

Our results suggest that gene-for-gene interactions are widespread in the natural pathosystem consisting of *C. ipomoeae* and its *Ipomoea* hosts. These species thus constitute a promising system for investigating the evolution of gene-for-gene interactions.

## Materials and Methods

### Ethics Statement

No specific permits were required for the collections used in these experiments. Some collections were made in public road right-of-ways, and where collections were made from private land, permission to do so was granted by landowners. No endangered or protected species were affected by our fieldwork.

### Species

*Ipomoea coccinea*, *I. hederacea*, and *I. purpurea* are annual plants commonly found in agricultural field margins in the southeastern United States, and are commonly infected by the rust fungus *Coleosporium ipomoeae*. In nature where *C. ipomoeae* is present in often co-occurring populations of these species, plants are either infected by the rust, or uninfected and showing signs of gross or microscopic hypersensitive response [27].

*Coleosporium ipomoeae* is a heteroecious rust pathogen that infects members of *Convolvulaceae*, including *Ipomoea*, as its alternate host in a clonal summer stage of its macrocyclic life cycle. Single rust strains are able to infect multiple species of *Ipomoea*. The primary hosts of *C. ipomoeae* belong to the genus *Pinus*, which are infected by the rust in the autumn [28].

### Combinations of host species/populations and rust isolates tested

Four combinations of host species/populations and rust isolates were used to determine the mode of inheritance of resistance (Table 1). Because rust spores can be dispersed long distances by the wind, all host species/populations used in these crosses have the potential to interact with all of the rust strains used. More specifically, the rationales for choosing each of these combinations are as follows:

1. Crosses 1 and 2 (Table 1A). These crosses involved a plant from one susceptible *I. purpurea* population, the rust strain collected from plants of that population, and a plant from a second *I. purpurea* population resistant to that rust strain. This type of comparison would be relevant to situation in which wind-dispersed spores of the rust strain were dispersed into the second, resistant population. These tests thus inquire about the genetic basis of pre-existing resistance to a novel rust genotype.
2. Crosses 3 and 4 (Table 1A). These two crosses involve rust strains collected from the same site as the resistant host species but from a different host species. It also involves a second population of the host species that is known to be susceptible to that strain. These tests thus inquire about the genetic basis of resistance to a rust strain that the resistant host population encounters naturally.
3. Cross 5 (Table 1B). This cross involves a resistant population of *I. hederacea* and a rust strain collected from a different host species at the same site. It also involves another population of *I. hederacea* that is resistant to this rust strain. Thus, this cross inquires about whether the pre-existing resistance in the second population involves the same gene as the resistance in the first population, which is naturally exposed to this rust strain.
4. Crosses 6 and 7 (Table 1B). Each of these crosses involves two populations of *I. purpurea* that are resistant to a rust strain from a third population of *I. purpurea*. The *I. purpurea* populations thus have pre-existing resistance to the tested rust strain. Each of these crosses also inquires about whether the same gene is involved in this pre-existing resistance for each pair of *I. purpurea* populations.

### Source Populations and Rust Collection

**Figure 1:**
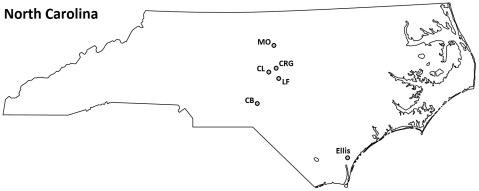
locations of host populations and rust accessions used in crossing experiments.

Seeds and rust spores were collected from populations of the three *Ipomoea* species in North Carolina (locations shown in Figure 1.) The specific seed and rust accessions are listed in Table 1. Host accessions are designated by locality and host species, *e.g.* CRG-P designates seeds collected from *Ipomoea purpurea* at locality CRG. Rust accessions are designated in similar fashion, *e.g.* LF-P indicates that rust spores were collected from *I. purpurea* plants from site LF. Specific populations were chosen based on prior cross-inoculation experiments that indicated that most or all host individuals in a given population were either susceptible or resistant to a particular rust strain.

Rust urediospores were collected from the field from infected leaves, which were removed from plants and placed in airtight bags for transport to the laboratory, where spores were washed with distilled water from live pustules into the reservoir of a sprayer. Spores were collected immediately before experimental inoculations. Because we could not collect all needed spores from a single lesion, and because single-spore isolates cannot be propagated successfully due to an apparent limit on the number of serial uredinial inoculations before inoculum loses ability to infect plants in the greenhouse, the spores for a given accession represent spores collected from several leaves on ten individuals of a given host species. While this means that a rust accession may consist of multiple genotypes, previous experiments indicated that an accession normally behaves as a single genotype with respect to virulence/avirulence. In particular, accessions from the same host at the same locality, but collected in different years, produce similar patterns of virulence across a panel of hosts [19]. While multiple genotypes could complicate our genetic analyses by introducing additional variability that could cause proportions of resistant and susceptible plants to deviate from those expected under single-gene inheritance, this problem did not arise in our experiments (see Results).

### Crosses

Under the standard gene-for-gene model, resistance to a particular pathogen strain is expected to be mediated by allelic variation at a single locus, with resistance dominant to susceptibility [29], [30]. To determine whether inheritance of resistance in *Ipomoea* is consistent with these expectations, we performed a series of crosses (Table 1A). In each cross, one parental individual was from a population that had previously been characterized as susceptible to a particular rust accession by cross-inoculation experiments, while the other parent was from a population that had previously been characterized as susceptible. The rust accessions were determined to be different in additional experiments undertaken to describe compatibility (data not shown). F_1_ individuals were selfed and approximately 120 selfed offspring (S_2_ individuals) were scored for resistance to that accession. Parental individuals were also scored for resistance. For each set of populations crossed, several pairs of parental individuals were crossed and their S_2_ descendents examined independently.

Because it was possible that a resistant parent might be heterozygous for resistance, we selfed each parent and scored 12 F_1_ offspring for resistance. Parents that produced some susceptible individuals were deemed heterozygous, and data involving these parents were discarded. By scoring 12 F_1_ individuals, the probability of falsely accepting a resistant parent as homozygous was 0.032.

To test whether different genes confer resistance in different populations, we performed allelism tests. For each test, we crossed two host populations that previous cross-inoculation experiments indicated were resistant to a given rust accession. The F_1_ individuals were selfed and S_2_ individuals were tested for resistance using the given rust accession (Table 1B). An average of 188 S_2_ individuals were tested for each of three crosses. In this type of test, we attempt to distinguish between two hypotheses: (1) both resistant populations carry the same resistance allele at the same locus, so that both have the genotype R_1_R_1_; (2) populations have resistance alleles at different loci, so that population 1 has genotype R_1_ R_1_ r_2_ r_2_, while population 2 has genotype r_1_ r_1_ R_2_ R_2_, where upper case denotes a resistant allele and lower case denotes a susceptible allele. If hypothesis (1) is correct, then resistance will not segregate in the F_2_ population and no susceptible F_2_ individuals will be produced. By contrast, if hypothesis (2) is correct, then some F_2_ individuals will be r_1_ r_1_ r_2_ r_2_ and thus will be susceptible. If susceptible individuals are found, hypothesis (1) can be rejected.

### Testing for resistance/susceptibility

Plants to be tested were grown from seed collected from the field. Seeds were germinated in potting soil in flats at the Duke University greenhouse, and then moved to a growth chamber in which they were watered every other day and experienced a 16-hour photoperiod and thermal regimen of 16 h at 32°C, 8 hours at 22°C. At plant age 21 days, plants were inoculated with an isolate of *C. ipomoeae* urediospores collected from the field.

For inoculation, soil and experimental plants were saturated with distilled water 8 hours prior to the onset of darkness in the growth chamber and flats were covered with 8″ clear plastic domes to elevate humidity and facilitate spore germination. Each flat contained four randomly-placed known susceptible plants used as positive controls, and 32 S_2_ plants from experimental crosses. 5 mL of uredinial inoculum suspension per flat was applied *via* a fine spray to the undersides of leaves. For two weeks after inoculation, plants were not watered or otherwise disturbed, and observed to detect the hypersensitive response (indicating resistance) or the presence of uredia (indicating susceptibility). A plant was scored as resistant if it exhibited a hypersensitive response and no uredia, where a plant was scored as susceptible if uredia were present. All 1361 plants scored in this experiment exhibited either uredia or a hypersensitive response, but not both.

### Data analysis

Our objective was to determine whether inheritance of resistance was consistent with the gene-for-gene expectation that among S_2_ individuals of a cross, resistant and susceptible individuals should occur in a 3∶1 ratio. Deviation from this ratio was assessed using maximum likelihood. In particular, we calculated the likelihood for two models, one in which the estimated ratio was unconstrained, and one in which the ratio was constrained to be 3∶1. Twice the log of the ratio (likelihood in unconstrained model)/(likelihood in constrained model) was used as the test statistic, which is distributed as χ^2^ with 1 degree of freedom. A significant value of the test statistic indicates a deviation from a 3∶1 ratio. S_2_ descendents from different parental pairs were tested for heterogeneity. Because there was no evidence of heterogeneity in any of the crosses, individuals from all pairs were then pooled to test for an overall deviation from the expected 3∶1 ratio.

The rationale underlying the allelism tests is that if in two resistant populations resistance alleles reside at the same locus, then the S_2_ individuals from a cross between the populations should never be susceptible. At the opposite extreme, if they reside at unlinked loci, then 1/16^th^ of the S_2_ individuals should be susceptible. Thus, the existence of any S_2_ susceptible individuals indicates non-allelism. In cases of non-allelism, the recombination fraction, r, between the loci was estimated from
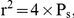
where P_s_ is the proportion of S_2_ individuals that are susceptible. Confidence intervals for r were estimated by calculating, for different possible r values, the likelihood ratio statistic for two likelihoods: L_1_ = likelihood of the observed numbers of resistant and susceptible individuals, given r; and L_2_, the likelihood of the observed numbers of resistant and susceptible individuals for the maximum likelihood estimate of r. The confidence interval then contains the set of r values for which this statistic is not significant.
